# Generation of blastoids from human parthenogenetic stem cells

**DOI:** 10.1093/lifemedi/lnad006

**Published:** 2023-02-18

**Authors:** Ke Zhong, Yu-Xin Luo, Dan Li, Zhe-Ying Min, Yong Fan, Yang Yu

**Affiliations:** Key Laboratory for Major Obstetric Diseases of Guangdong Province, The Third Affiliated Hospital of Guangzhou Medical University, Guangzhou 510150, China; Department of Obstetrics and Gynecology, Beijing Key Laboratory of Reproductive Endocrinology and Assisted Reproductive Technology and Key Laboratory of Assisted Reproduction, Ministry of Education, Center for Reproductive Medicine, Peking University Third Hospital, Beijing 100191, China; Department of Obstetrics and Gynecology, Beijing Key Laboratory of Reproductive Endocrinology and Assisted Reproductive Technology and Key Laboratory of Assisted Reproduction, Ministry of Education, Center for Reproductive Medicine, Peking University Third Hospital, Beijing 100191, China; National Clinical Research Center for Obstetrics and Gynecology, Beijing 100191, China; Clinical Stem Cell Research Center, Peking University Third Hospital, Beijing 100191, China; Key Laboratory for Major Obstetric Diseases of Guangdong Province, The Third Affiliated Hospital of Guangzhou Medical University, Guangzhou 510150, China; Key Laboratory for Major Obstetric Diseases of Guangdong Province, The Third Affiliated Hospital of Guangzhou Medical University, Guangzhou 510150, China; Department of Obstetrics and Gynecology, Beijing Key Laboratory of Reproductive Endocrinology and Assisted Reproductive Technology and Key Laboratory of Assisted Reproduction, Ministry of Education, Center for Reproductive Medicine, Peking University Third Hospital, Beijing 100191, China; National Clinical Research Center for Obstetrics and Gynecology, Beijing 100191, China; Clinical Stem Cell Research Center, Peking University Third Hospital, Beijing 100191, China

**Keywords:** human parthenogenetic ESCs, blastoids, hEPSCs, imprinted genes, X chromosome

## Abstract

Parthenogenetic embryos derive their genomes entirely from the maternal genome and lack paternal imprint patterns. Many achievements have been made in the study of genomic imprinting using human parthenogenetic embryonic stem cells (hPg-ESCs). However, due to developmental defects and ethical limits, a comprehensive understanding of parthenogenetic embryonic development is still lacking. Here, we generated parthenogenetic blastoids (hPg-EPSCs blastoids) from hPg-ESC-derived extended pluripotent stem cells (hPg-EPSCs) using our previously published two-step induction protocol. Morphology, specific marker expression and single-cell transcriptome analysis showed that hPg-EPSCs blastoids contain crucial cell lineages similar to blastoids (hBp-EPSCs blastoids) generated from human biparental EPSCs (hBp-EPSCs). Single-cell RNA-seq compared the expression of genes related to imprinting and X chromosome inactivation in hPg-EPSCs blastoids and hBp-EPSCs blastoids. In conclusion, we generated parthenogenetic blastoids, which will potentially promote the study of genomic imprinting in embryonic development and uncover the influence of parental origin bias on human development and pathological mechanisms.

## Introduction

Mammals lack the ability of unisexual reproduction by parthenogenesis [1, 2]. In humans, natural parthenogenesis by spontaneous activation of an unfertilized oocyte results in ovarian teratoma [3]. *In vitro*, artificial parthenogenetic activation of mammalian oocytes through electric stimulation and chemical agents can obtain parthenogenetic embryos. Human parthenogenetic embryonic stem cells (hPg-ESCs) can be obtained from the inner cell mass (ICM) of parthenogenetic embryos [4–6]. hPg-ESCs have typical human embryonic stem cell (hESC) morphology and express key pluripotent transcription factors and specific cell surface markers. hPg-ESCs also have functions comparable to those of human biparental ESCs (hBp-ESCs, the ESCs derived from normal embryos). Moreover, hPg-ESCs possess the potential to differentiate into the derivatives of three embryonic germ layers *in vitro* by embryonic body formation, and *in vivo* by teratomas formation [5, 7].

The consequences of changes in genomic imprinting during mouse development are well understood. In some studies, parthenogenetic and androgenetic cells were transplanted into mouse embryos to form chimeras [8]. The spatial distribution and differentiation potential of parthenogenetic and androgenetic cells were measured. Parthenogenetic cells failed to form trophectoderm (TE) and primordial endoderm (PrE), and parthenogenetic embryos arrested by mid-gestation and contained embryonic tissues but poorly developed extraembryonic tissues [9]. Developmental failure was also observed in human uniparental embryos. Although mice as models can help explain human genomic imprinting, the majority of annotated imprinted genes are not shared between species [10], and these species-specific patterns of imprinted genes may have unique effects on human diseases and development [11]. X chromosome inactivation (XCI) also differs greatly between mouse and human. Ethical issues limit research on the effects of genomic imprinting on human embryos, and the development of human parthenogenetic embryos is rarely studied. In recent years, with the increasingly strict regulation of embryos, the exploration of human embryogenesis has been severely limited, and human blastoids are regarded as a substitute for studying human development. Several research groups have recently generated various human blastoids [12–17], whose cell sources and construction technologies and materials are gradually enriched. The construction of human blastoids derived from human parthenogenetic extended pluripotent stem cells (hPg-EPSCs) can be used to study the function of imprinted genes during embryonic development. Since the genetic material of hPg-ESCs is derived solely from the maternal genome, parthenogenetic blastoids are also a conducive tool for exploring the effect of parental origin bias on human diseases.

Here, we attempted to generate blastoids from hPg-EPSCs by a two-step induction protocol and then analyzed the morphology and molecular characteristics of hPg-EPSCs blastoids. The hPg-EPSCs blastoids will shed light on the role of imprinted genes in the development of parthenogenetic embryos and the underlying mechanisms of human development and diseases.

## Results

### Characteristics identification of hPg-ESCs and hPg-EPSCs

**Figure 1.**
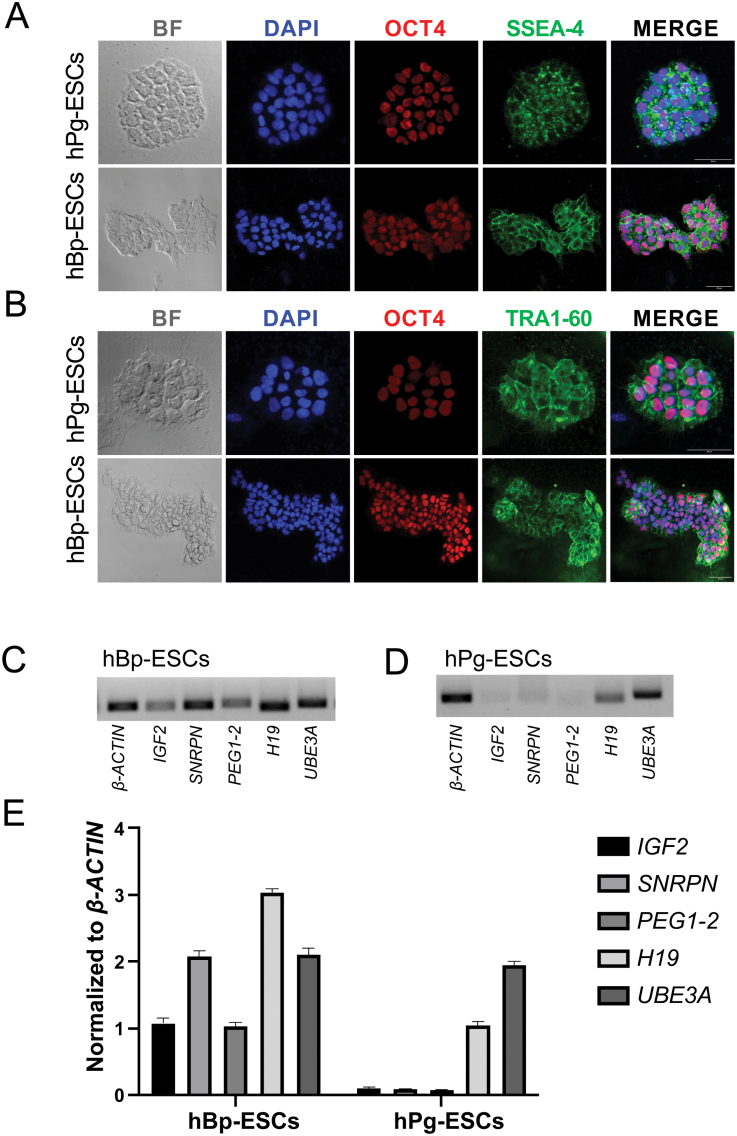
**Charateristics analysis of hPg-ESCs and hBp-ESCs.** Undifferentiated colonies of hPg-ESCs and hBp-ESCs on mouse feeder layer cells, expressed pluripotent markers, OCT4 (A) and SOX2 (B), and cell surface markers SSEA-4 (A) and TRA1-60 (B). Scale bar = 50 μm. RT‒PCR analysis of the expression of imprinted genes in hBp-ESCs (C) and hPg-ESCs (D). From left to right, lines are the results for the following genes in the order of *β-ACTIN*, *IGF2*, *SNRPN*, *PEG1-2*, *H19*, and *UBE3A*. (E) qRT‒PCR analysis of the expression profiling of imprinted genes in hPg-ESCs and hBp-ESCs.

In previous study, we have derived hPg-ESCs from parthenogenetic embryos [7]. We detected the pluripotent state and parthenogenetic origin of these thawed cells in this study. hPg-ESCs colonies exhibited typical stem cell morphology, which was compact, flattened, had a high nuclear to cytoplasmic ratio and contained distinct nuclei. In addition, the positive expression of conventional pluripotent markers, OCT4 and SOX2 and cell surface markers, SSEA-4 and TRA1-60 were also detected in hPg-ESCs (Fig. 1A and 1B). Then, we identified their parthenogenetic origin according to the expression of imprinted genes. Imprinted genes display parent-of-origin dependent monoallelic expression. To verify the imprinting status of hPg-ESCs, we performed PCR to analyze the expression of human imprinted genes in both hPg-ESCs and hBp-ESCs. hBp-ESCs derived from human natural embryos were used as a control. RT‒PCR results showed that the paternally expressed genes *SNRPN*, *PEG1-2* [6, 18], and *IGF2* were not expressed, while the maternally expressed genes *H19* and *UBE3A* were expressed in hPg-ESCs (Fig. 1C and 1D). In hBp-ESCs, paternally and maternally imprinted genes were both expressed. The qRT‒PCR results showed similar relative expression levels of imprinted gene expression in both of them (Fig. 1E).

Next, we converted hPg-ESCs to hPg-EPSCs using N2B27-LCDM media with a monolayer of feeder cells. After long-term culture, hPg-ESCs maintained their morphology and viability. Moreover, hPg-EPSCs were demonstrated to have the ability to form colonies by single-cell passaging. Additionally, we reverted primed hBp-ESCs to hBp-EPSCs using the same method. The induced morphologies and processes were similar to those of hPg-ESCs.

### Human parthenogenetic blastoids generated from hPg-EPSCs

Recently, many studies have been performed to establish blastoids using human pluripotent stem cells (hPSCs) to mimic early human pre- and post-implantation embryonic development, however, the generation of hPg-EPSCs blastoids is still lacking.

**Figure 2.**
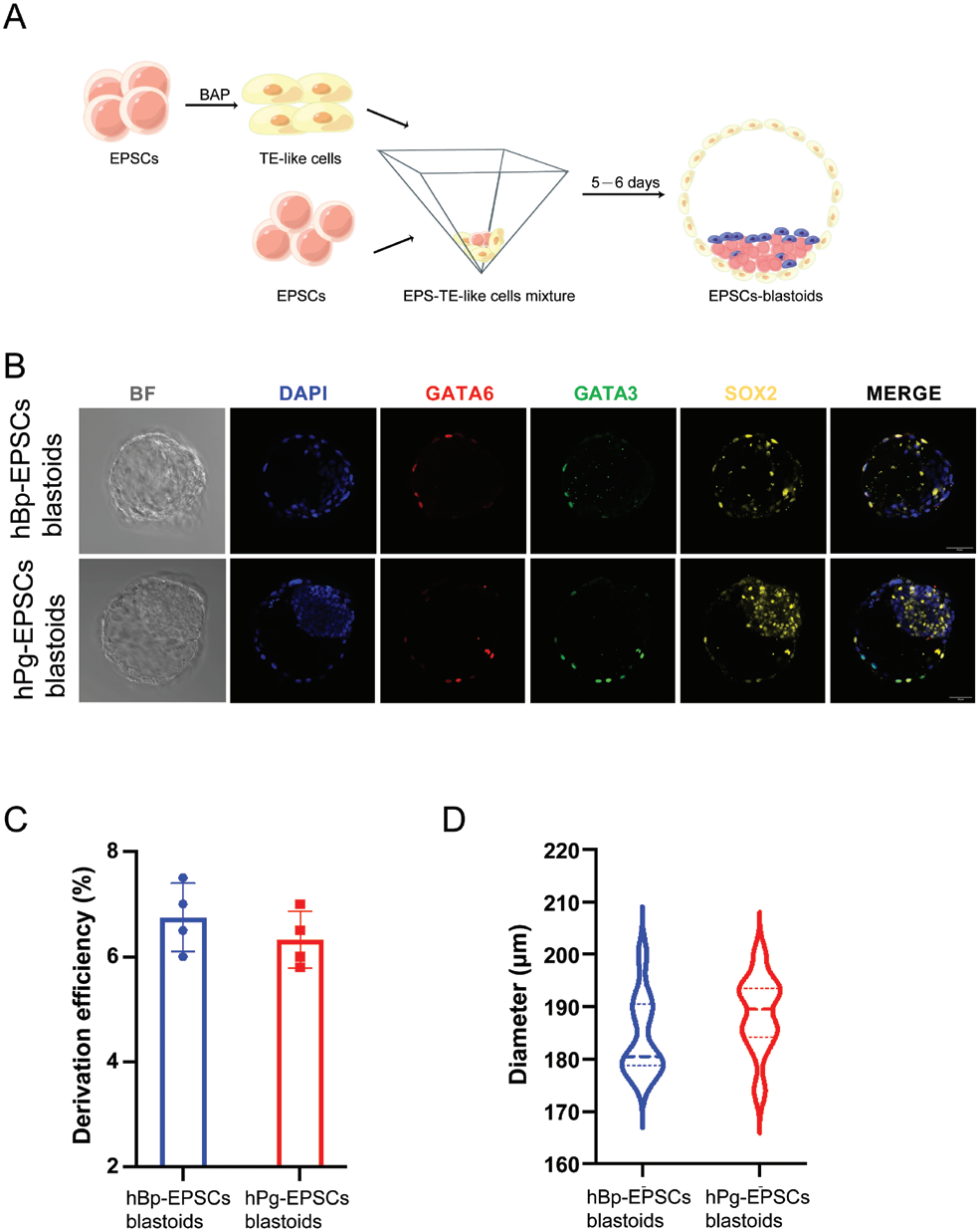
**Induction of human parthenogenetic blastoids under 3D conditions.** (A) Schematic of EPSCs-blastoids formation. EPSCs were first induced to TE-like cells with BAP, and then TE-like cells were mixed with EPSCs and seeded together in 3D condition on Day 0. The aggregates further differentiated and developed into EPSCs blastoids. (B) Immunofluorescence staining of hPg-EPSCs blastoids and hBp-EPSCs blastoids for the EPI lineage marker (SOX2), TE lineage markers (GATA3), and PrE lineage marker (GATA6). Scale bar = 50 μm. (C) Derivation efficiency was comparable between hPg-EPSCs blastoids and hBp-EPSCs blastoids. (D) Mean diameter was comparable between hPg-EPSCs blastoids and hBp-EPSCs blastoids.

Minor modified from published methods [12, 19], we pretreated hPg-EPSCs and hBp-EPSCs with BMP4, A83-01, and PD173074 (BAP) for approximately 3 days to induce TE-like cells to enhance the tendency of TE differentiation (Fig. 2A). Then, these induced cells were mixed with hPg-EPSCs or hBp-EPSCs, individually, and seeded in 3D microwells. After 24 h, cells in most of the microwells formed aggregates. By days 5–6, human blastoids formed some aggregates, which exhibited typical blastocyst morphology.

Immunofluorescence was performed on hPg-EPSCs and hBp-EPSCs to check whether these blastoids had epiblast (EPI), PrE and TE lineages corresponding to natural blastocysts. The outer layer corresponding to cells in days 5–6 blastoids expressed GATA3 (TE specific gene), while inner cells had high expression of SOX2 (EPI specific gene), surrounded by the surrounding cells with GATA6 (PrE-specific gene) expression (Fig. 2B). However, the PrE and TE markers in hPg-EPSCs blastoids had less expression than EPI markers. In the hBp-EPSCs blastoids, three lineage markers were expressed, and the expression of GATA6 and GATA3, the lineage markers of PrE or TE, individually, were slightly more oblivious than that in the hPg-EPSCs blastoids (Fig. 2B). Overall, hPg-EPSCs blastoids showed similar efficiency and morphology compared with hBp-EPSCs blastoids (Fig. 2C and 2D).

Taken together, these results indicated that hPg-EPSCs blastoids contained TE and ICM/EPI lineages and that a part of them had three cell lineages corresponding to late blastocysts.

### Single-cell transcriptome analysis of hPg-EPSCs blastoids

**Figure 3.**
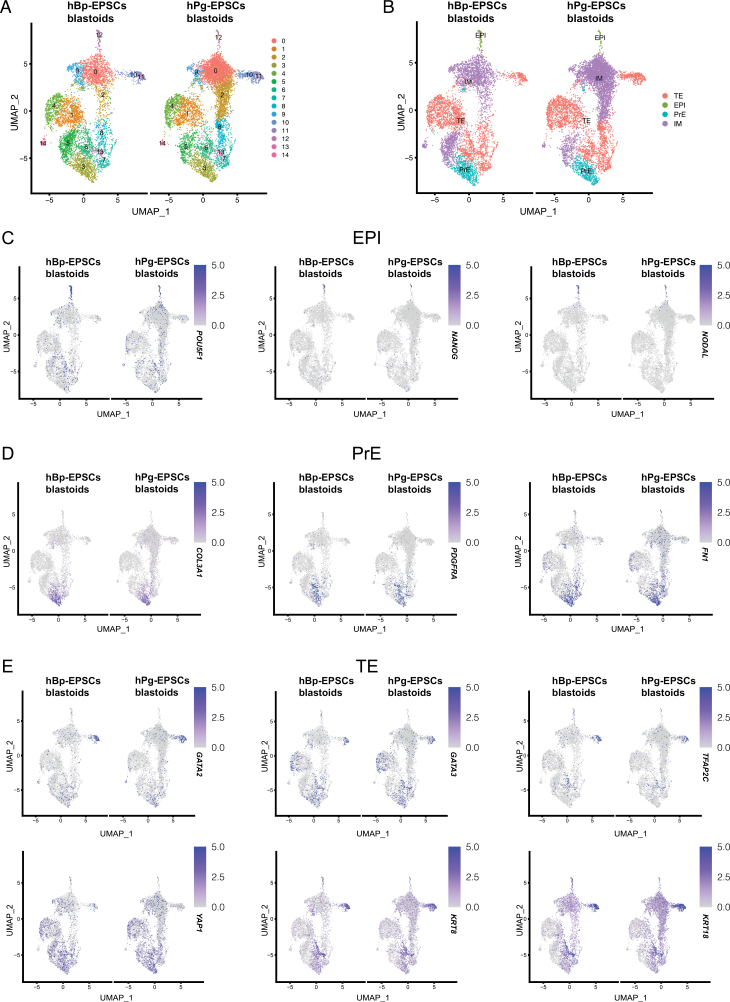
**Landscape of the transcriptome in human parthenogenetic and biparental blastoids on day 6.** (A) The UMAP plot of single cells from hPg-EPSCs blastoids and hBp-EPSCs blastoids showed that the cells in blastoids were divided into 14 clusters on day 6. (B) After bioinformatic analysis, the cells in blastoids were divided into 4 major clusters on day 6. The EPI/ICM, PrE, TE, and IM subgroups, which were determined according to lineage-specific markers. The cells in IM subgroups expressed all three lineage markers or some uncertain genes. (C–E) The expression of representative lineage-specific genes of hPg-EPSCs blastoids and hBp-EPSCs blastoids on day 6 shown in the UMAP plot, including ICM/EPI (C), PrE (D), and TE markers (E).

To further investigate hPg-EPSCs blastoids at the molecular level, we performed single-cell RNA-seq (scRNA-seq) analysis on hPg-EPSCs blastoids. Uniform manifold approximation and projection (UMAP) clustering analysis revealed that both hPg-EPSCs blastoids and hBp-EPSCs blastoids could be divided into 14 clusters (Fig. 3A), and these clusters could be further divided into 4 major clusters on day 6. The EPI/ICM, PrE, TE, and intermediate (IM) subgroups were determined according to lineage-specific markers (Fig. 3B). The cells in IM subgroups expressed all three lineage markers or some uncertain genes. Similar to hBp-EPSCs blastoids, in the hPg-EPSCs blastoids, some clusters not only expressed EPI markers, such as *OCT4* and *NANOG* (Fig. 3C), or PrE markers, such as *PDGFRA*, *FN1*, and *COL1A3* (Fig. 3D) but also co-expressed TE markers, such as *GATA2*, *GATA3*, *YAP*, and *TFAP2C* (Fig. 3E). Based on the expression of specific genes, we characterized three clusters as ICM/EPI, two clusters as PrE, and three clusters as TE. Moreover, we compared scRNA-seq data from hPg-EPSCs blastoids with hBp-EPSCs blastoids, and they were similar. In conclusion, our scRNA-seq analysis revealed that hPg-EPSCs blastoids contained the three cell lineages in natural blastocysts.

### Parthenogenetic features explored in the hPg-EPSCs blastoids

**Figure 4.**
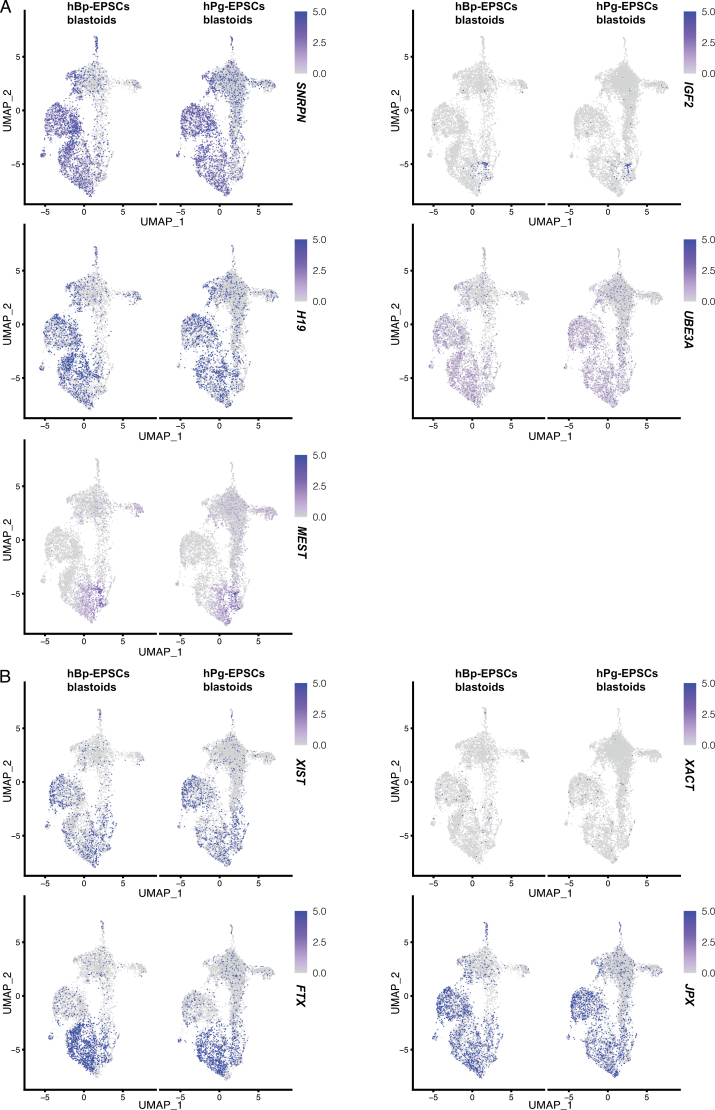
**Parthenogenetic features of hPg-EPSCs blastoids.** (A) The expression of representative imprinted genes of hPg-EPSCs blastoids and hBp-EPSCs blastoids on day 6 shown in the UMAP plot, including *SNRPN*, *MEST*, *IGF2*, *H19*, and *UBE3A.* (B) The expression of representative XCI-related genes of hPg-EPSCs blastoids and hBp-EPSCs blastoids on day 6 shown in the UMAP plot, including *XIST*, *XACT*, *FTX*, and *JPX*.

Provided that the imprinting analysis in human blastocysts has not been comprehensively explored, we detected the expression of imprinted genes in hPg-EPSCs blastoids and hBp-EPSCs blastoids. There were no significant differences in the expression of either the maternally imprinted genes or the paternally imprinted genes found between both of them (Fig. 4A). Moreover, considering the unique characteristics of the paternal genome, we examined the status of X chromosome. A previous study showed that the XCI status of some hPg-ESCs was unstable [20], making them susceptible to culture conditions and freezing processes, suggesting that parental genomic imprinting has an impact on the genomic stability of offspring. However, unlike the mechanism of XCI in mouse, the expression level of X-inactive specific transcript (*XIST*) alone cannot reflect the status of the X chromosome, and X-active coating transcript (*XACT*) is reported to be involved in XCI in human. We explored the expression levels of *XIST*, *XACT*, and several X chromosome linked genes [21, 22]. The results showed that the expression of *XIST* was high in some clusters, while the expression of *XACT* was low in most clusters. Some regulators of *XIST* expression in mouse were also analyzed in hPg-EPSCs blastoids, including *JPX* [23, 24] and *FTX* [25], noncoding genes in mouse characterized as X-inactivation center (XIC)-linked *XIST* activators [26]. Both of them have orthologues in human, but their role is still elusive. Investigation of hESCs in naïve and primed states showed that *JPX* was involved in *XIST* expression [27]. Similar to the expression of *XIST*, *JPX*, and *FTX* were expressed in some clusters and had relatively high expression levels (Fig. 4B). Moreover, we detected the expression of some X chromosome linked genes in hPg-EPSCs blastoids and hBp-EPSCs blastoids (Fig. S1). Similarly, there were no significant differences between both of them.

## Discussion

Comparing the efficiency of hPg-EPSCs blastoids and hBp-EPSCs blastoids, there was no significant difference between them. Moreover, hBp-EPSCs blastoids had similar morphology to those derived from hPg-EPSCs. Transcriptome analysis showed that hPg-EPSCs blastoids had some intermediate clusters and that the expression of lineage markers was low and even unable to distinguish among different clusters, which were also observed in hBp-EPSCs blastoids.

We have proven the parthenogenetic origin of hPg-ESCs and detected the expression levels of maternally and paternally imprinted genes in hPg-EPSCs blastoids. The results of hPg-ESCs showed typical bimaternal imprinting patterns, which was similar to previous reports [5, 7]. However, some studies have indicated that the loss of genomic imprinting is specific to naïve PSCs [28]. Considering the similar characteristics of hPg-EPSCs and naïve PSCs, hPg-EPSCs may gradually lose genomic imprinting during culture, which suggests unstable genomic imprinting.

hPg-ESCs have been reported to have genetic instability and epigenetic abnormalities. X chromosome microdeletion was observed in hPg-ESCs following long-term culture [20]. Dose compensation through the inactivation of one of X chromosomes, termed XCI, in female mammals is a key event for compensating for the imbalance of X chromosome genes in males and females. XCI is triggered by the long noncoding RNA, *XIST*. XCI plays an important role in cell biological processes and embryonic development, and abnormal XCI can also lead to some diseases [29]. Our knowledge of the early developmental dynamics of the X chromosome mainly comes from mouse. Although XCI is highly conserved, there are also species differences. In human, *XIST* is expressed on both X chromosomes of parental origin [30–32], and both X chromosome genes are expressed [33] despite the accumulation of *XIST* in female preimplantation embryos. However, the total expression amount decreased, called X chromosome dampening (XCD) [30], which may obtain the dose compensation effect. At the same time, the number of active X chromosomes in the supernumerary X chromosomes was independent of the parental origin of the X chromosome [34], and the XCI statuses of hPg-ESCs and hBp-ESCs were similar [20], suggesting that XCI was independent of the parental origin. In mouse, the accumulation of *XIST* on the X chromosome drove the removal of active histone modification and the formation of heterochromatin markers, such as tri-methylation of H3 lysine 9 (H3K9me3) and tri-methylation of H3 lysine 27 (H3K27me3), which completed the heterochromatization of X chromosome [35]. *XIST* was expressed in human preimplantation development, but lack of heterochromatin marks [30] may be responsible for the stable expression of X chromosome genes. Moreover, some studies reported evidences against XCD and found that the combination of XCI and upregulation of the active X chromosome achieved dosage compensation [21]. To further assess the status and timing of XCI in early human development, more research models and methods are required. XCI has been reported to be a random choice in the placenta [36], but RNA-sequencing and methylation analysis results showed a bias towards inactivating paternal X chromosome [37]. However, in human development, the dynamics and mechanisms of XCI are greatly unknown. By using the blastoid model generated by hPg-ESCs, we can detect XCI and the chromatin landscape of *XIST*-coated active chromosomes in early human development, which will be very helpful to explore the mechanisms and regulatory networks of XCI.

In conclusion, we induced hPg-EPSCs from hPg-ESCs to differentiate into blastoids using an *in vitro* 3D system. The obtained blastoids partially recapitulated the early development of natural blastocysts in morphology. Additionally, the effect of parthenogenetic conditions on blastoids formation was confirmed by comparing the hPg-EPSCs balstoids and hBp-EPSCs blastoids. hPg-EPSCs blastoids could be used as a high-throughput model for parthenogenetic studies to explore the effects of genomic imprinting on embryonic development and related diseases and to further explore the mechanisms of X chromosome linked gene dosage compensation.

## Research limitations

Although we explored parthenogenetic features, including the expression of imprinted genes and XCI, in hPg-EPSCs blastoids, no significantly differences were detected between hPg-EPSCs blastoids and hBp-EPSCs blastoids. We speculated that the induction system and cell resource might be suboptimal and thus the differences between hPg-EPSCs blastoids and hBp-EPSCs blastoids were weakened. Therefore, further studies should be explored the underlying parthenogenetic features.

## Materials and methods

### Human samples and ethics statement

hPg-ESCs and hBp-ESCs were derived in the lab of Peking University Third Hospital and published in the previous studies [7]. The present study was approved by the Institutional Review Board of Peking University Third Hospital (S2020022).

### Generation of human EPSCs from primed PSCs

hPg-ESCs, hBp-ESCs, hPg-EPSCs, and hBp-EPSCs were cultured at 37°C, 5% CO_2_ and saturated humidity. These cell lines were all cultured on mitomycin C-treated mouse embryonic fibroblast (also the feeder cells). hPg-ESCs and hBp-ESCs were maintained in DMEM/F12 (Thermo Fisher Scientific, 11330-032) supplemented with 20% KNOCKOUT SR Medium (Thermo Fisher Scientific, 10828-028), 1% Penicillin-Streptomycin (Thermo Fisher Scientific, 15140-122), 2 mM l-GlutaMAX (Thermo Fisher Scientific, 35050-061), 0.1 mM MEM NEAA (Thermo Fisher Scientific, 11140-050), 0.1 mM 2-Mercaptoethanol (Thermo Fisher Scientific, 21985-023), and 10 ng/mL recombinant bFGF (Thermo Fisher Scientific, PHG0261). According to the manufacturers’ directions, hPg-ESCs and hBp-ESCs were cultured in N2B27-LCDM medium to convert to hEPSCs. N2B27 basal medium consisted of 1:1 mixture of DMEM/F12 (Thermo Fisher Scientific, 11330-032) and Neurobasal Medium (Thermo Fisher Scientific, 21103-049) supplemented with 0.5X N2 supplement (Thermo Fisher Scientific, 17502-048), 0.5X B27 supplement (Thermo Fisher Scientific, 12587-010), 1% Penicillin-Streptomycin (Thermo Fisher Scientific, 15140-122), 1% MEM NEAA (Thermo Fisher Scientific, 11140-050), 1% l-GlutaMAX (Thermo Fisher Scientific, 35050-061), 5% KNOCKOUT SR Medium (Thermo Fisher Scientific, 10828-028), and 0.1 mM 2-Mercaptoethanol (Thermo Fisher Scientific, 21985-023). To prepare the N2B27-LCDM medium, small molecules and cytokines were added into the N2B27 basal medium as indicated at the following final concentrations: 10 ng/mL recombinant human LIF (L, 10 ng/mL; Peprotech, 300-05), CHIR 99021 (C, human: 1 mM, mouse: 3 mM; Tocris, 4423), (S)-(+)-dimethindene maleate (D, 2 mM; Tocris, 1425), and minocycline hydrochloride (M, 2 mM; Santa Cruz Biotechnology, sc-203339). hPg-ESCs and hBp-ESCs were digested into single cells by TrypLE Express (Thermo Fisher Scientific, 12604-021) and cultured on feeder cells. After 12 h of seeding, the human ESC medium was replaced with N2B27-LCDM medium. The N2B27-LCDM medium was changed every day. Dome-shaped colonies emerged, which were picked manually and transferred to a fresh feeder cell-coated dish. Cells were routinely passaged every 3 or 4 days. All cell lines routinely tested negative for mycoplasma.

### Derivation of hPg- or hBp-EPSCs blastoids under 3D conditions

The blastoids were induced and cultured in 37°C, 5% O_2_, 5% CO_2_, and saturated humidity. First, hPg-EPSCs were induced with BMP4, A83-01, PD173074 and Y-27632 (BAP) for 3–4 days to differentiate into TE-like cells. BAP differentiation medium was composed of N2B27 basal medium supplemented with 10 ng/mL BMP4 (R&D Systems, 314-BP-010), 1 μM A83-01 (Tocris, 2939), 0.1 μM PD173074 (Selleckchem, S1264), and 10 μM Y-27632 (Selleckchem, S1049). On the first day, marked as day 0, the cultured hPg-EPSCs or hBp-EPSCs were dissociated into single cells with TrypLE Express (Thermo Fisher Scientific), and the feeder cells were removed by pasting twice on 0.5% gelatin (Sigma) for 30 min. hPg-EPSCs or hBp-EPSCs were seeded into a dish pretreated with Matrigel and were induced in BAP differentiation medium. After 3 days of differentiation, cells were dissociated into single cells treated with TrypLE Express. Then, the BAP-treated cells (8 × 10^4^ cells) and hPg-EPSCs or hBp-EPSCs (~ 2 × 10^4^ cells) were mixed together at a 4:1 ratio to a total of 1 × 10^5^ cells per well with 0.5 mL culture medium and seeded into one well of a 24-well AggreWell 400 culture plate that was pretreated with Anti-Adherence Rinsing Solution (Stem Cell Technologies, #07010). The culture medium was slightly changed daily. On day 6, aggregates were picked and collected. The culture medium of human EPSCs blastoids consists of N2B27-LCDM medium and IVC1 medium at a ratio of 1.5:1 (v/v). IVC1 culture medium was prepared as follows: advanced DMEM/F12 (Thermo Fisher Scientific, 12634-010), 20% heat-inactivated FBS (Thermo Fisher Scientific, 30044-333), 0.5% penicillin/streptomycin (Thermo Fisher Scientific, 15140-122), 2 mM l-GlutaMAX (Thermo Fisher Scientific, 35050-061), 1% Insulin-Transferrin-Selenium-Ethanolamine (ITS-X) (Thermo Fisher Scientific, 51500-056), 1% Sodium Pyruvate (Thermo Fisher Scientific, 11360-070), 8 nM β-Estradiol (Sigma‒Aldrich, E8875), 25 μM N-acetyl-l-cysteine (Sigma‒Aldrich, A7250), and 200 ng/mL Progesterone (Sigma‒Aldrich, P0130).

### Analysis of imprinted genes

Total RNA was extracted using an RNA extraction kit (Magen, R4011-02). RNA (1 μg) was reverse-transcribed to cDNA using Maxima™ h minus cDNA Synthesis Master Mix (Thermo Fisher Scientific, M1662) in a 20 μL reaction volume. Then, cDNA (1 μg) was subjected to PCR amplification with primers for β*-ACTIN*, *IGF-2*, *SNRPN*, *PEG1-2*, *H19*, and *UBE3A* using Taq DNA polymerase. For qPCR, cDNA (1 μg) was subjected to quantitative PCR amplification using PowerUp SYBR Green Master Mix (Applied Biosystems, A25741). The samples were run in technical triplicates. The sequences of the primers and PCR conditions were shown in Table S1.

The PCR products were analyzed by 1% polyacrylamide gel electrophoresis. qPCR was performed in Applied Biosystems QuantStudio3. The results were analyzed by the comparative ΔΔCt method. The expression of β*-ACTIN* served as a control.

### Immunofluorescence staining

Cells and blastoids were fixed with 4% paraformaldehyde (Nakalai Tesque) in phosphate-buffered saline (PBS) for 15–30 min at room temperature, and then samples were permeabilized with 0.2% Triton X-100 in PBS for 30 min at room temperature. The samples were blocked with 5% BSA in PBS for 2 h at room temperature. Next, samples were incubated with primary antibodies diluted in fresh blocking buffer (5% BSA in PBS) overnight at 4°C. The next day, the samples were washed 3 times for 5–10 min with PBST (PBS containing 0.1% Tween-20). Fluorescence-conjugated secondary antibodies diluted in blocking buffer were diluted and incubated at temperature for 2 h, and the samples were washed 3 times for 10 min each time with PBST.

Zeiss LSM 710 confocal microscope was used for imaging. Images were processed by ZEN (Zeiss) and Fiji (ImageJ, V2.0.0) software. The primary antibodies and dilutions were as follows: mouse anti-OCT4 (Santa Cruz, sc5279, 1:200), mouse anti-SOX2 (Abcam, ab171380, 1:200), mouse anti-GATA3 (R&D Systems, 1:100), and goat anti-OCT4 (Santa Cruz, 1:100). The secondary antibodies were Alexa Fluor 488 goat anti-rabbit IgG (H+L) (Thermo Fisher Scientific, A-11008), Alexa Fluor 488 donkey anti-rabbit (H+L) (Thermo Fisher Scientific, A-21206), Alexa Fluor 555 goat anti-mouse IgG (H+L) (Cell Signaling Technology, 4409S), Alexa Fluor 647 donkey anti-goat (H+L) (Thermo Fisher Scientific, A-21447), and Alexa Fluor 647 goat anti-rabbit IgG (H+L) (Abcam, ab150083, GR3269213).

### scRNA-seq

hPg-EPSCs blastoids and hBp-EPSCs blastoids were manually picked up using a mouthpipette and washed 3 times. Approximately 150 hPg-EPSCs blastoids or hBp-EPSCs blastoids were harvested and dissociated with a homemade enzyme mix composed of 0.5X versene (Lonza, 17711E), 0.5X Acumax (Innovative Cell Tech, AM105), and 0.05X Dnase I (STEMCELL Tech, 07900) at 37°C for 30 min with agitation. Dissociated cells were spun down and washed with PBS containing 0.05% BSA 3 times and resuspended in the same buffer. Cell density was determined by a TC10 cell counter (Bio-Rad, 1450001). Dissociated cells (~6000 cells for hPg-EPSCs blastoids and ~8000 cells for hBp-EPSCs blastoids) were loaded into the Chromium single-cell controller (10× Genomics) to generate single-cell gel beads in the emulsion according to the manufacturer’s protocol. The library was generated using Chromium Single Cell 3ʹ Reagent Kits according to the manufacturer’s manual. The two libraries were pooled and sequenced using Nextseq 500 (150 cycles, high output) (CapitalBio Technology, China).

### Analysis of scRNA-seq data

Alignment, filtering, barcode counting, and UMI counting were performed with the Cell Ranger count module to generate a feature-barcode matrix and to determine clusters. The R package Seurat 4.0.3 was used to analyze the feature-barcode matrix as follows: first, cells whose gene numbers were <100, unique features count over 60,000, and mitochondrial gene ratio was more than 20% according to quality control matrix plots were regarded as abnormal and filtered out. Then, UMI counts were normalized with the NormalizeData function using default settings. Nonlinear dimensionality reduction was clustered with resolution setting at 0.6, and visualized by UMAP.

### Statistical analysis

Statistical analyses were performed with GraphPad Prism 8 (GraphPad Software, California, USA), using unpaired two-tailed Student’s *t*-tests. All of the statistical tests performed are indicated in the figure legends. The data are presented as mean ± SD, and *P* < 0.05 was regarded as significant differences.

### Data availability

Single-cell RNA-seq data have been deposited in the Gene Expression Omnibus (GEO) under assession number GSE213648 (scRNA-seq data website: https://www.ncbi.nlm.nih.gov/geo/query/acc.cgi?acc=GSE213648).

## Supplementary Material

lnad006_suppl_Supplementary_Material
