# Supplementary material for: Generation of blastoids from human parthenogenetic stem cells
Source: Life Med. 2023 Feb 18;2(1):lnad006. doi: 10.1093/lifemedi/lnad006 (PMC11748981; doi:10.1093/lifemedi/lnad006)
Supplement: lnad006_suppl_Supplementary_Material [file lnad006_suppl_Supplementary_Material.pdf]

Supplementary Materials

Generation of blastoids from human parthenogenetic stem cells

Figure S1. Expression profiling of X chromosome linked genes.

The expression of representative X chromosome linked genes of hPg-EPSCs blastoids and hBp-EPSCs blastoids on Day 6 shown in the UMAP plot, including *APOO*, *ARTX*, *HNRNPH2*, *RBMX2*, *VBP1*, *SYAP1*, and *EIF2S3*.

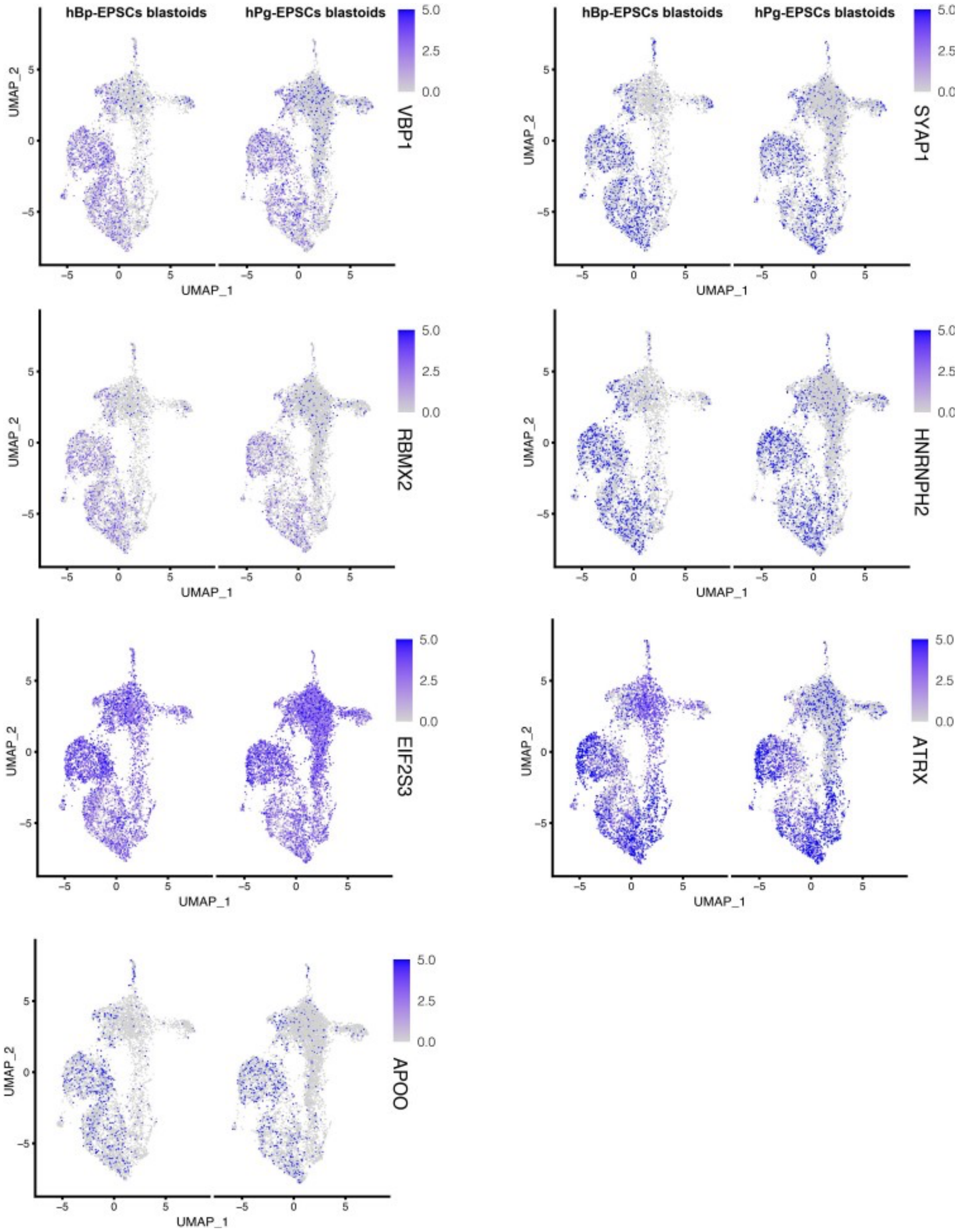

Table S1. PCR primers used to detect gene expression in hPg-ESCs and hBp-ESCs

Gene	Sense (5'-3')	Antisense (5'-3')	Size (bp)
β-ACTIN	AGGGCAGTGATCTCCTTCTG	ATTGGCAATGAGCGGTTCCG	212
IGF-2	CTCTCCGTGCTGTTCTCTCC	CGGGCCAGATGTTGTACTTT	196
SNRPN	CTTCTGCCCAGCTTGCAT	TGAAGATTCGGCCATCTTGC	205
PEG1-2	GCTGCTGGCCAGCTCTGCAC GGCTG	CTTGCCTGAAGACTTCCATGA GTGA	230
H19	TACAACCACTGCACTACCTG	TGGCCATGAAGATGGAGTCG	148
UBE3A	TTGCCACCATTTGTAGACCA	CCAATTTCTCCCTTCCTTCC-3	224
